# Lupin (*Lupinus spp*.) seeds exert anthelmintic activity associated with their alkaloid content

**DOI:** 10.1038/s41598-019-45654-6

**Published:** 2019-06-21

**Authors:** O. Dubois, C. Allanic, C. L. Charvet, F. Guégnard, H. Février, I. Théry-Koné, J. Cortet, C. Koch, F. Bouvier, T. Fassier, D. Marcon, J. B. Magnin-Robert, N. Peineau, E. Courtot, C. Huau, A. Meynadier, C. Enguehard-Gueiffier, C. Neveu, L. Boudesocque-Delaye, G. Sallé

**Affiliations:** 1 UMR1282 Infectiologie et Santé Publique, INRA, Université de Tours, F-37380 Nouzilly, France; 2 EA 7502 SIMBA, Université de Tours, Faculté de Pharmacie, F-37000 Tours, France; 3 UE332 La Sapinière, INRA, F-18174 Osmoy, France; 4 Agroécologie, AgroSup Dijon, INRA, Univ. Bourgogne, Univ. Bourgogne Franche-Comté, F-21000 Dijon, France; 5 Département Physiologie Animale, Université de Tours, Faculté des Sciences et Techniques, F-37000 Tours, France; 6 GenPhySE, INRA, Université de Toulouse, INPT, ENVT, F-31326 Castanet-Tolosan, France

**Keywords:** Parasite biology, Phenotypic screening

## Abstract

The growing range of drug resistant parasitic nematode populations threatens the sustainability of ruminant farming worldwide. In this context, nutraceuticals, animal feed that provides necessary dietary requirements while ensuring parasite control, could contribute to increase farming sustainability in developed and low resource settings. In this study, we evaluated the anthelmintic potential of lupin seed extracts against the major ruminant trichostrongylids, *Haemonchus contortus* and *Teladorsagia circumcincta*. *In vitro* observations showed that seed extracts from commercially available lupin varieties could significantly but moderately inhibit larval migration. This anthelmintic effect was mediated by the seed alkaloid content and was potent against both fully susceptible and multidrug resistant *H*. *contortus* isolates as well as a susceptible *T*. *circumcincta* isolate. Analytical chemistry revealed a set of four lupanine and sparteine-derivatives with anthelmintic activity, and electrophysiology assays on recombinant nematode acetylcholine receptors suggested an antagonistic mode of action for lupin alkaloids. An *in vivo* trial in *H*. *contortus* infected lupin-fed ewes and goats failed to demonstrate any direct anthelmintic effect of crude lupin seeds but infected lupin-fed goats suffered significantly less parasite-mediated blood losses. Altogether, our findings suggest that the anthelmintic potential of lupin remains limited. However, the potent alkaloids identified could lead to the development of novel drugs or may be used in combination with current anthelmintics to improve their efficacy.

## Introduction

Anthelmintic resistance is a major issue for the sustainable management of both human-^1^ and livestock-infective helminth species^2–4^. Gastro-intestinal parasitic nematodes (GIN) significantly impact human development, amounting to a loss of 10 million disability-adjusted life years^5^ and major production losses in the livestock industry^6,7^. While novel control solutions are urgently needed, only a few anthelmintic compounds have been released in the recent years, and some, like monepantel^8^, rapidly developd drug resistance in the field^9^. Vaccines remain difficult to design^10^ and experimental evidence suggest that GIN show enough transcriptomic plasticity to circumvent the vaccine-induced response of their host^11^. In the veterinary setting, the selective breeding of more resistant strains^12,13^, targeted-specific treatment approaches^14^, and the use of tannin-rich plant extracts^15,16^ have been studied. This latter strategy relies on the combined properties of certain forage species with good nutritional properties and bioactive compounds, known as “nutraceuticals”^15^. This approach will limit drug use, lower drug selection pressure and can be deployed under low resource settings^16^.

Lupin (*Lupinus sp*.*)* is a grain legume that belongs to the genistoid clade of the Fabaceae family^17^. It produces protein- and energy-rich seeds used for ruminants^18^ or laying hens^19^ feed, and contributes to reduce risk of obesity, diabetes and cardiovascular diseases in humans^20^. These interesting nutritional properties are however counterbalanced by quinolizidine and piperidine alkaloids that confer both bitterness and toxicity to the alkaloid-rich lupin varieties^21^. In mammals, these alkaloids block the excitatory neuro-transmission through the binding of nicotinic acetylcholine receptors, nAChRs^21–23^ and also act as antagonists of muscarinic acetylcholine receptors^23^. As a result, ingestion of alkaloid-rich varieties can lead to acute cholinergic toxicity^24^ and subacute toxicity has been reported in livestock pregnant females leading to abnormal foetal development^25^. To prevent toxicity, regulations in Australian and some European countries impose that lupin seeds should contain no more than 0.02% of alkaloids^26,27^ and genetic selection program have established commercial varieties with low alkaloid content^27^. This dual property of lupin seeds could be leveraged for the control of GIN in livestock based on the fact that GIN nAChRs are well characterized pharmacological targets for the control of parasitic nematodes^28^. These transmembrane ligand-gated ion channels are made of five subunits that associate together to form homo- or heteropentameric receptors^28^. Widely used anthelmintics *i*.*e*. levamisole^29^, pyrantel^30^, and monepantel^31^, are agonists of these receptors, whereas derquantel, a derivative from the oxindole alkaloid paraherquamide^32^, acts as a nAChR antagonist^33^. Lupin quinolizidine alkaloids show insect toxicity^34^, previous findings in helminths of veterinary or medical importance^35–38^ and plant-parasitic nematodes^39,40^ have demonstrated the anthelmintic potential of plant extracted alkaloids. Lupin is known to also lower the soil burden of the vine parasitic nematode *Xiphinema index*^41^. Therefore, the quinolizidine alkaloids contained in lupin seed may display some anthelmintic activity and provide novel compounds for GIN control. In addition, the residual alkaloid content contained in commercially available alkaloid-poor lupin varieties may exert a beneficial effect in parasite infected livestock. In such a case, lupin seed could be used as a nutraceutical for grazing ruminants.

To investigate these two hypotheses, we first combined *in vitro* assays on major parasitic trichostrongylids exposed to lupin seed extracts with analytical chemistry to characterize the anthelmintic properties of lupin extracts. Second, an i*n vivo* trial with commercial lupin seed in growing ewe and goats was implemented to determine whether lupin could serve as a nutraceutical.

## Results

### Lupin seed extracts show anthelmintic effect on *H*. *contortus* infective larvae

**Figure 1:**
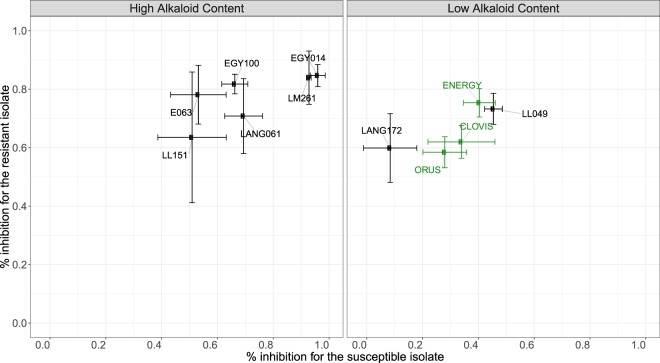
Initial screening highlights ENERGY and E063 as the most potent varieties. Figure depicts the average percentage of larval migration inhibition (relative to negative control) measured on drug-susceptible (x-axis) and drug-resistant (benzimidazole, levamisole, ivermectin; y-axis) *Haemonchus contortus* isolates exposed to 11 lupin varieties seed total extracts (used at a concentration of 5 mg/mL). Lines show standard deviation measured from three replicates. Green dots stand suitable for commercially available varieties.

As a first step, aqueous extracts from 11 alkaloid-rich and -poor lupin seeds (Supplementary Table 1) were tested against drug-susceptible and multidrug-resistant *H*. *contortus* infective larvae using a larval migration assay and at a concentration of 5 mg/mL (Fig. 1, Supplementary Table 2). Every aqueous extract considered (with the exception of LANG172) exerted a significant reduction of larval migration across parasite isolates whatever its anthelmintic resistance status (Fig. 1, Supplementary Table 2). Aqueous extracts from alkaloid-rich seeds demonstrated a 77.7% inhibition of larval migration and were thus generally more potent than the alkaloid-poor varieties (27.1% ± 0.04% difference in average inhibition, *F*_*1*,*63*_ = 28.68, *P* < 10^−4^; Fig. 1, Supplementary Table 2). Among alkaloid-poor varieties, extracts from ENERGY and LL049 exerted similar reductions in migration across parasite isolates (57.8% ± 0.2% and 59.3% ± 0.16% respectively) and significantly out-performed other alkaloid-poor varieties (16.8% ± 0.03% difference, *F*_*2*,*27*_ = 62.8, *P* < 10^−4^, Fig. 1).

In an initial attempt to establish the contribution of alkaloids to this inhibitory effect, *H*. *contortus* larvae were exposed to the sole alkaloid fraction from alkaloid-rich varieties. The potency of alkaloid fractions (Supplementary Table 3) was minimal for LL151 in the fully susceptible *H*. *contortus* isolate (52% ± 2.77% s.d.) and the strongest effect was observed for E063 on the multidrug resistant *H*. *contortus* isolate (82% ± 3.58% s.d.).

After this initial screening, the anthelmintic potential of lupin seed extracts was demonstrated against both drug-susceptible and multidrug-resistant isolates. One alkaloid-rich and alkaloid-poor were selected for further study. The ENERGY variety was retained as the most potent commercial variety that could eventually be used as a nutraceutical. The E063 variety was further considered as an alkaloid-rich control given the highest inhibitory effect of its alkaloidic fraction.

### Lupin alkaloids are more potent than non-alkaloid compounds across isolates and nematode species

To further characterize the lupin seed inhibitory effect on parasitic nematodes, total seed extracts were fractionated into alkaloidic and non-alkaloidic fractions for both E063 and ENERGY varieties. Alkaloids accounted for 3.30% of the E063 seed mass whereas the alkaloid-poor ENERGY seed alkaloid content amounted 0.043% of the seed mass.

**Figure 2:**
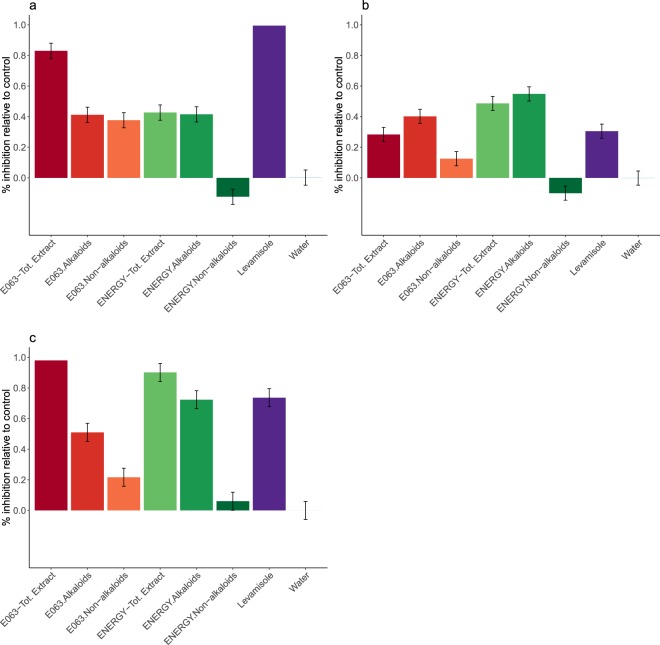
Comparative inhibitory potential of ENERGY and E063 lupin seed total extract, alkaloidic and non-alkaloidic fractions. Picture depicts observed inhibitory effects of lupine seed extracts (expressed as the percentage of observed migration relative to negative control) on drug susceptible (**a**), multidrug resistant *H*. *contortus* (**b**) and susceptible *T*. *circumcincta* (**c**) infective larvae migration after exposure to different solutions. “Tot. Extract” stands for total seed extract. Levamisole (10 µM) and water were used as positive and negative controls respectively, and lyophilized extracts were used at a concentration of 5 mg/mL. Each condition was run across six replicates.

The larval migration assay performed with each extract revealed that alkaloids of both varieties significantly inhibited the larval migration in comparison to the negative control across drug-resistance status or nematode species (Fig. 2, supplementary Table 4). Non-alkaloid compounds from ENERGY had no effect on larval migration (12.5% ± 0.06% inhibition difference relative to control, Student’s t-test *t*_39_ = −2.15, *P* = 0.38), whereas the E063 non-alkaloidic fraction was as potent as the alkaloid fraction against susceptible *H*. *contortus* larvae (Fig. 2a, supplementary Table 4). E063 total seed extract could inhibit the susceptible *H*. *contortus* L3 with the same magnitude as the 10 µM levamisole solution (99.3% ± 0.06% and 82.8% ± 0.06% mean inhibition for levamisole and E063 total extract respectively, *z-score* = −2.84, *P* = 0.09).

Alkaloids recovered from ENERGY were significantly more potent than levamisole at inhibiting the migration of the resistant isolate (24.3% ± 0.07% inhibition difference, *z-score* = 3.72, *P* = 5.1 × 10^−3^, Fig. 2b, supplementary Table 4).

Lupin seed total extracts and alkaloid fractions also significantly inhibited *Teladorsagia circumcincta* infective larvae (Fig. 2c, supplementary Table 4).

Altogether these results confirmed the potential of ENERGY and E063 alkaloids as potent anthelmintics against major trichostrongylid of small ruminants and their ability to control multidrug-resistant *H*. *contortus* isolates.

### Alkaloids of ENERGY and E063 lupin varieties are different and do not exert the same anthelmintic activity

**Figure 3:**
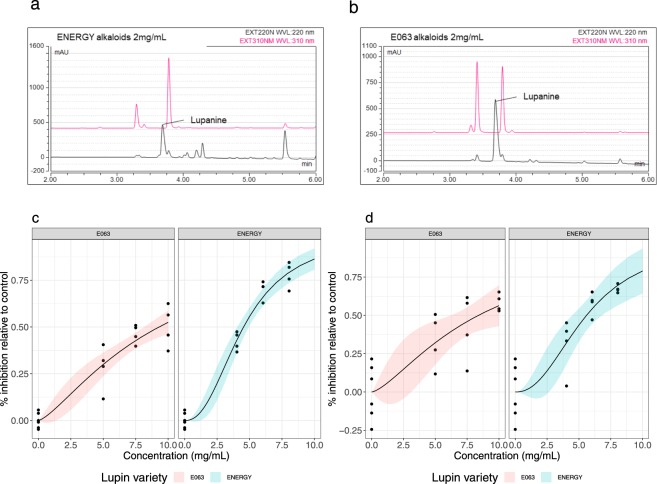
ENERGY and E063 alkaloids differ in their content and anthelmintic effect. HPLC profiles measured at 220 nm (black) and 310 nm (pink) reveal a more diverse alkaloidic content (spikes in profile) in ENERGY (**a**) than in E063 (**b**). Bottom panels depict the inhibited fraction of larvae relative to control for alkaloid concentration ranging between 0 and 10 mg/mL, for a drug susceptible (**c**) and a multidrug resistant (**d**) isolates. Solid line stands for the fitted log-logistic regression curve and shaded area indicates 95% confidence interval (red for E063 and blue for ENERGY).

To investigate the difference between E063 and ENERGY alkaloids, chromatographic analyses of their respective alkaloid fractions were performed (Fig. 3a). Lupanine was the most abundant alkaloid in both cases (21.5% and 49.5% for ENERGY and E063 respectively, supplementary Table 5). ENERGY alkaloids exhibited a more complex alkaloid profile (five major alkaloids identified) compated to E063 alkaloids (Fig. 3a).

Importantly, the qualitative differences in alkaloid profiles between both varieties were also associated with contrasted inhibitory effects: estimated ENERGY alkaloids IC_50_ was at least 2 mg/mL lower than for E063 alkaloids across *H*. *contortus* isolates (differences in IC_50_ between varieties of 4.76 mg/mL, *P* < 10^−4^ and 2.79 mg/mL, *P* = 0.03 for the drug-susceptible and resistant isolate respectively; Fig. 3b, supplementary Table 6).

To further delineate the inhibitory effect of alkaloids, a concentration response assay of lupanine, *i*.*e*. the major lupin alkaloid, was implemented against the susceptible *H*. *contortus* isolate (Supplementary Fig. 1). While infective larvae responded in a dose-response fashion after levamisole exposure (IC_50_ = 1.61 µM), the lupanine-induced inhibition increased in a linear fashion with lupanine concentration, was highly variable and inhibited less than 25% of the control migration (Supplementary Fig. 1).

These observations would favour a more potent alkaloid mixture in the ENERGY seeds than in the E063 variety. They also suggest non-selective toxic effects of lupaninewhich cannot explain the observed anthelmintic activity..

### Identification of alkaloidic compounds and evaluation of their anthelmintic activity

As lupanine, the most abundant lupin alkaloid, was not the major cause of the observed anthelmintic effects in the ENERGY alkaloids extract, a Centrifugal Partition Chromatography (CPC) pH zone refining process was used to identify active compounds (supplementary Fig. 2). Ten simplified fractions were obtained. Initial screening of their potencies against *H*. *contortus* revealed four highly active fractions (7 to 10) that could inhibit between 61.2 ± 5.2% and 93.6% ± 3.7% of larval migration of both drug-susceptible and multidrug-resistant isolates (supplementary Table 7). HPLC analyses indicated that lupanine was not found in any of these four fractions.

**Figure 4:**
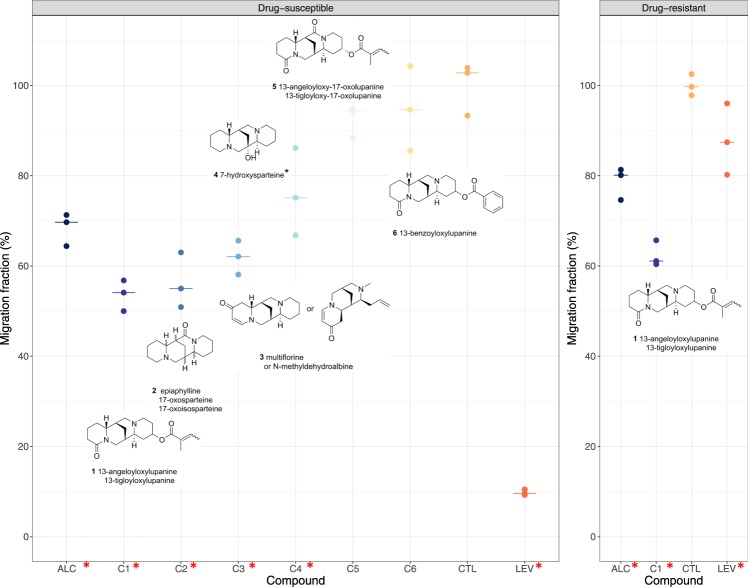
Identified alkaloids and their anthelmintic activity against *H*. *contortus* infective larvae. Plotted are the results of the Automated Larval Migration Assay expressed as a percentage of drug-susceptible or drug-resistant *H*. *contortus* larvae migrating (relative to negative control; CTL; water) after exposure to the whole alkaloid extract (ALC, 5 mg/mL), six alkaloidic compounds (**C1** to **C6**; 150 µg/mL for compound **2** and 250 µg/mL else) or appropriate negative (CTL) and positive control (LEV; levamisole 10 µM). Inferred compound structures are provided next to each plot. Bars represent the median migration fraction and red asterisks indicate compound with significant effects.

The alkaloids contained in these four fractions (7 to 10) were further purified using semi-preparative HPLC (Supplementary Table 5) that yielded six pure alkaloids (compounds **1** to **6**; Fig. 4, Supplementary Fig. 3). Because of the limited amount of compound available, their anthelmintic activity was tested using an Automated Larval Migration Assay (ALMA) at concentrations ranging between 250 and 150 µg/mL (Fig. 4). Using this assay, total alkaloid extract demonstrated a significant (31 and 21% inhibition, Fig. 4) but weaker effect than that measured with LMIA (Supplementary Table 4). Among the six tested compounds, compounds **5** and **6** did not significantly inhibit drug-susceptible *H*. *contortus* larval migration (*P = *0.2 and 0.5, Fig. 4). Larval migration after exposure to other compounds was significantly inhibited falling to 53.6% ± 3.42% s.d. (compounds **1**) to 76% ± 9.68% s.d. (compounds **4**) of control larval migration (*P* = 0.05, Fig. 4). Compound **1** was also a significant inhibitor of multidrug-resistant larvae which reduced their migration to 62.4% ± 2.88% s.d. (Fig. 4).

LC-ESI-MS analysis was performed to identify compound structures by comparison with the literature. Compound **1** was identified as 13-angeloyloxy or 13-tigloyloxy-lupanine (Fig. 4). Compounds **2** and **4** were identified as sparteine-derivatives, *i*.*e*. an oxidated analogue of sparteine (epiaphylline, 17-oxosparteine or 17-oxoisosparteine) and 7-hydroxysparteine respectively (Fig. 4). The structure of compound **3** structure was compatible with multiflorane or N-Methyldehydroalbine (Fig. 4).

Our combined approach using analytical chemistry and the recently developed ALMA to analyse ENERGY seed extracts revealed lupanine- and sparteine-derivatives as promising novel anthelmintics.

### Electrophysiology shows that lupin alkaloids are antagonists of nematode nicotinic acetylcholine receptors

**Figure 5:**
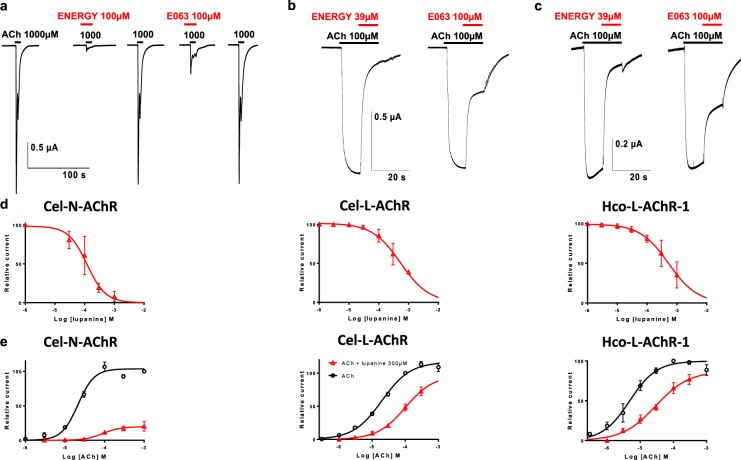
Inhibitory effect of lupin seed extracts from ENERGY and E063 varieties and lupanine on nematode acetylcholine receptor (AChR) subtypes expressed in *Xenopus laevis* oocytes. Figure shows representative recording traces from the *Caenorhabditis elegans* nicotine-sensitive AChR (**a**) and levamisole-sensitive AChR (**b**), and the *H*. *contortus* levamisole-sensitive AChR (**c**) after exposure to acetylcholine (ACh) alone or in the presence of ENERGY and E063 extracts. Concentrations (μM) are indicated above each trace. Bars indicate the time period of the drug application (ACh in black and lupin alkaloids in red). Panel d depicts lupanine concentration-response inhibition curves obtained on Cel-N-AChR (left), Cel-L-AChR (middle) and Hco-L-AChR-1 (right). Panel e displays acetylcholine concentration-response curves either alone (in black), or with 300 µM lupanine (in red) on Cel-N-AChR (left), Cel-L-AChR (middle) and Hco-L-AChR-1 (right).

To elucidate the anthelmintic mechanisms of lupin alkaloids, we investigated their interactions with AChRs. We expressed the recombinant AChRs, *H*. *contortus* and *Caenorhabditis elegans* levamisole receptors (Hco-L-AChR-1, Cel-L-AChR respectively), and the nicotine-sensitive *C*. *elegans* receptor (Cel-N-AChR) in *Xenopus laevis* oocytes and perfused them with ENERGY and E063 extracts as well as lupanine, the most abundant alkaloid in lupin extracts (Fig. 5, Supplementary Table 8).

The application of lupin seed extracts to the oocytes expressing either Hco-L-AChR-1, Cel-L-AChR or Cel-N-AChR did not induce any currents thus ruling out a direct agonist effect. However, pre-incubation of *X*. *laevis* oocytes with ENERGY and E063 extracts significantly blocked the subsequent acetylcholine-elicited currents for every receptor subtype (Fig. 5a,b). The inhibition of ACh-induced current was significantly higher with ENERGY alkaloids than with E063 alkaloids for both Cel-L-AChR (84 ± 1.9% s.d. versus 59 ± 9.3% s.d., *P* = 0.03, n = 6; Fig. 5b) and Hco-L-AChR-1 (91 ± 4% s.d. versus 65 ± 9.6% s.d., *P* = 0.03, n = 6; Fig. 5c). The magnitude of this effect was more reduced for Cel-N-AChR (87.5 ± 11.2% s.d. versus 64.3 ± 23.7% s.d., *P* = 0.06 n = 4; Fig. 5a). These antagonistic effects were completely reversible after subsequent washing (Fig. 5a).

Because of their limited respective amounts, it was not possible to undertake both ALMA and electrophysiology measurements with alkaloid compounds **1** to **4**. Lupanine is the main alkaloid in both investigated lupin varieties. Despite its lack of a direct anthelmintic effect, it was considered further for electrophysiology study to gather insights on the mode of action of its derivatives. Concentration-responses assay demonstrated that Cel-N-AChR was more sensitive to the lupanine inhibitory effect on ACh-elicited currents (IC_50_ = 116.5 ± 9.7 µM; Fig. 5d, Supplementary Fig. 4 and Table 8) whereas a similar inhibitory potential of this alkaloid was observed on levamisole- and nicotine-sensitive receptors (IC_50_ = 539.9 ± 90.2 µM and 548.8 ± 64.1 µM for Hco-L-AChR-1 and Cel-L-AChR respectively; Fig. 5d, Supplementary Fig. 4 and Table 8).

Lupanine-mediated antagonism was further investigated by comparing ACh concentration-responses in the absence and in the presence of 300 µM lupanine (Fig. 5e, Supplementary Table 8). Parallel dose-response plots were obtained with a shift in ACh EC_50_ that increased by 3.71 to 5.95times its value in the presence of lupanine for Hco-L-AChR-1 (EC_50[ACh]_ = 4.8 ± 0.5 µM versus EC_50[ACh+300µM Lup]_ = 28.6 ± 8.4 µM, *P* = 0.005, *t* = −2.83) and for Cel-N-AChR (EC_50[ACh] = _21.7 ± 0.9 µM versus EC_50[ACh+300µM Lup]_ = 80.6 ± 27.5 µM; *P* = 0.034, *t* = −2.14). This feature is typical of competitive antagonism.The same tendency was observed for Cel-N-AChR but was not significant (EC_50[ACh] = _19.6 ± 1.7 µM versus EC_50[ACh+300µM Lup] = _99.1 ± 47.8 µM; *P* = 0.1, *t* = −1.66; Fig. 5e). Finally, we observed an 81% drop-off in the maximum amplitude of ACh-elicited currents for the Cel-N-AChR whereas this maximal current was less affected (38% decrease) for levamisole-sensitive receptors (Fig. 5e). This would be in favour of a non-competitive antagonism.

Altogether these results suggested that the anthelmintic potency of ENERGY is mediated by an non-competitive antagonistic mode of action of its alkaloid content. Electrophysiology also revealed striking differences between the sensitivity of nicotine-sensitive AChR subtypes to lupanine that may underpin the inhibitory potential of lupin extracts against the multidrug-resistant isolate.

### *In vivo* test on ewe lambs and dairy goats

**Figure 6:**
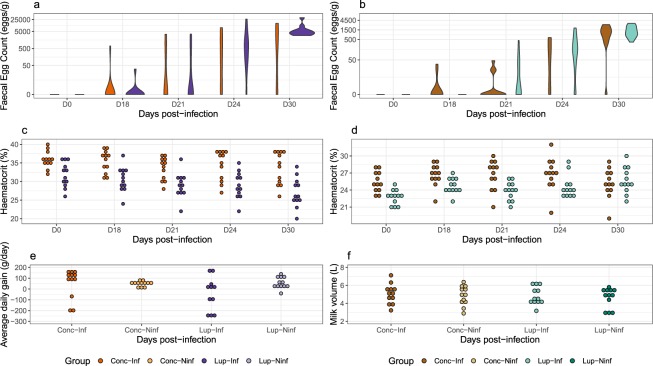
*In vivo* test of lupin energy seed on sheep and goats challenged with *H*. *contortus*. Figure depicts measured Faecal Egg Counts (**a**,**b**), haematocrit (**c**,**d**) and production traits (**e**,**f**) for growing ewes (**a**,**c**,**e**) and dairy goats (**b**,**d**,**f**). Average daily gain is the growth difference between 30 and 0 dpi scaled by the number of days. Milk volume is the sum of three recorded milk yields at 0, 21 and 30 dpi. Dots are coloured by experimental groups (Lup-Inf: lupin-fed and infected; Lup-Ninf: lupin-fed and not infected; Conc-Inf: concentrate-fed and infected; Conc-Ninf: concentrate-fed and not infected.

The last step of our study was to establish whether ENERGY crude seeds could be used as a nutraceutical. Homogeneous groups of growing sheep and dairy goats were subjected to experimental *H*. *contortus* infection (Supplementary Table 9) and fed with either supplementary lupin or control standard diet (Supplementary Table 10). The quantity of lupin in the diets (250 g/day and 450 g/day for ewes and goats respectively, Supplementary Table 10) was maximized within the recommended limits for animal feed protein and energy requirements. Faecal Egg Counts (FEC, Fig. 6a,b) increased throughout the experiment and reached maximal values at 30 days post-infection (dpi) and parasitic-mediated blood losses decreased haematocrit values (1.83% ± 0.45 and 1.5% ± 0.44 differences relatively to 18 dpi for ewes and for goats across conditions, Fig. 6c,d).

FEC did not show significant differences between lupin-fed and concentrate-fed animals suggesting lupin seed did not affect *H*. *contortus* within its host (*P* = 0.84, ${\chi }_{1}^{2}$ = 0.039 for ewes; *P* = 0.55, ${\chi }_{1}^{2}$ = 0.36 for goats; Fig. 5a,b). On the contrary, ewes fed with lupin had higher FEC values at 30 dpi than their concentrate-fed counterparts (116 eggs/g difference between groups, *P* = 2 × 10^−3^, *t*_66_ = 3.2; Fig. 6a). Also, lupin seeds did not delay the onset of egg excretion in sheep or goats and did not alter the larval development rate (Supplementary Table 11).

Similar haematocrits were measured between ewes of both group throughout the challenge (*P* = 0.10, ${\chi }_{1}^{2}$ = 2.68; Fig. 6b). However, goats exhibited a slightly different pattern: the overall trend in blood losses was not significantly different between diets (*P* = 0.75, ${\chi }_{1}^{2}$ = 0.10; Fig. 6c) but blood losses were significantly reduced in goats fed with lupin at 30 dpi (2.41% ± 0.63 difference in haematocrit between both groups, *P* = 3 × 10^−4^, *t*_66_ = 3.86; Fig. 6c). This would suggest an increased resilience in dairy goats fed with lupin seeds. But this increased resilience was not observed for production traits. Growth rate deteriorated in infected ewes fed with lupin seeds (−122 g/day 46.1, P = 0.01, *t*_3_ = −2.644; Fig. 6e) and goat milk yield remained unchanged between groups throughout the experimental challenge (*P* = 0.83, *F*_*3*,*44*_ = 0.3; Fig. 6e).

Despite the promising compounds harboured in ENERGY lupin seeds, these observations suggest that commercial lupin seed cannot be used to control major *H*. *contortus* infection in growing ewes at the dose tested in this setting. Still, it may contribute to dampen blood losses suffered by infected goats.

## Discussion

Our observations provide the first evaluation of the anthelmintic properties of lupin seed extracts against parasitic nematodes. *In vitro* assays revealed that the seed alkaloid content was potent against drug susceptible and multidrug resistant *H*.*contortus* and a similar effect was found in another trichostrongylid species. Electrophysiology data indicated that alkaloid effect could result from both competitive and non-competitive inhibition of parasitic nematode acetylcholine receptors. Analytical chemistry of the most potent alkaloid fractions revealed four lupanine- and sparteine-derivativeswhose potencies suggest they would underpin lupin anthelmintic effect. However, *in vivo* trials failed to demonstrate a direct anthelmintic effect of lupin seeds in infected ewes and goats. But infected dairy goats fed with lupin displayed lower blood losses.

Previous *in vitro* assays have already demonstrated the potency of plant extracted alkaloids against various parasitic helminths, i.e. *Schistosoma mansoni*^35,37^, *Trichuris muris*^35^ or *H*. *contortus*^38^. Reported potencies of pure compounds were generally in the range of 6 to 10 µg/ml^35,37,38^. This is in strong contrast with the much higher (2 mg/ml) estimated lupin alkaloid IC_50_. However, pure lupanine and sparteine derivatives could paralyse drug-suscepible and drug-resistant *H*. *contortus* larvae at a much lower concentration (150–250 µg/ml). Although it was not possible to estimate the IC_50_ of these compounds, these data suggest that some of the lupin alkaloids display stronger anthelmintic activity than the total alkaloid fraction. Their limited amount however remains a significant hurdle for better characterization of their anthelmintic effect and their affinity for GIN nAChRs.

This latter property is of primary importance for the design of novel drug that should have higher affinity for the parasite targets and lower toxicity in the host. Indeed, lupin alkaloids can induce a wide range of toxic effect in mammals^21^. Their IC_50_ on mammalian nAChRs can be as low as 0.14 µM for cytisine^23^ or 5 µM or lupanine^23^ and our data suggest that lupanine affinity for nematode nAChRs is lower. While the four lupanine- and sparteine-derivative compounds with higher anthelmintic activity may display lower IC_50_ on nematode receptors we would still need to ensure that they are not responsible for fetal toxicity in pregnant females as is the case for piperidine alkaloids^25^.

Electrophysiology of recombinant nematode receptors in *Xenopus* oocytes provide evidence for the molecular mechanism of the inhibitory effects of lupin seed extract. While lupanine exerted similar antagonistic effects against cholinergic receptors, in contrast, concentration-response curves suggested that lupanine acts differently with levamisole- and nicotine-sensitive receptor subtypes. Of note, EC_50_ shifts observed for levamisole-sensitive receptors would suggest a reversible competitive antagonist mode of action, whereas currents measured on Cel-N-AChR suggests that lupanine acts as a non-competitive antagonist. As these cholinergic receptors control muscle cell contractions at neuromuscular junctions and lupine seed extracts act upon several receptor subtypes the development is less likely. This underscores the importance of nAChRs for the development of novel anthelmintic compounds^42^ and highlights the need to investigate the putative modulatory effect of lupin alkaloids on other nematode ligand-gated ion channel subtypes.

Resolving the contribution of AChRs antagonism to the observed anthelmintic effects would require additional experiments that are beyond the scope of this study. Future work using RNA interference knock-down of acetylcholine receptor expression^43^ in parasitic larvae before further exposure to lupin alkaloids may elucidate the mechanisms involved. Nonetheless, our results indicated that the E063 enriched alkaloidic fraction was not necessarily exerting as much inhibition as the total seed extract and that significant larval migration inhibition was associated with the non-alkaloid seed extract in that case. This suggests that lupin seed extracts may contain non-alkaloid components which have additional effects on parasitic larvae. For example, non-alkaloid compounds, like flavonoids, could prevent alkaloid metabolism by the parasite as this is the case with ivermectin^44^. This would explain the relatively low effect observed for lupanine on larval migration in contrast with its clear antagonistic effect on cholinergic receptors.

Results from the *in vivo* experiment did not validate the nutraceutical potential of commercial lupin seeds. Unlike alkaloid-rich lupin varieties, commercial lupin seeds contain only traces of alkaloids^27^ thereby making lupin seeds palatable enough for livestock species^18,19^ as observed in our trial on ewes and goats. However, no direct effect on parasite infection load was observed and lupin-fed ewes had higher FEC than their control match. Despite the lack of beneficial effects in ewes and the similar egg excretion levels monitored between diets, blood losses were lessened in goats at the end of their challenge. Lupin omega-6 fatty acids content might underpin a beneficial pro-clotting effect as seen in hens^45^. However, the residual alkaloid content harboured in commercial lupin seeds does not seem to be sufficient to exert any direct anthelmintic effect, especially in ewes which received less seeds in this trial because of their reduced digestive capacity. Additional developments are hence required to deliver lupin-based solution to breeders. Selection of lupin varieties with higher alkaloid contents added to livestock diets could be an option as well as the combination of lupin seeds with tannin-rich forages.

In conclusion, we provided the first evidence of lupin seed anthelmintic properties against major trichostrongylid of ruminants and identified lupanine- and sparteine-derivatives as the likely active compounds. Our results also suggested that alkaloid compounds act as antagonist of parasitic cholinergic receptors. However, commercial lupin seeds did not contribute to reduced parasite egg excretion in experimentally challenged ruminants. More importantly, Alkaloid compounds harboured in commercial lupin seed could provide a foundation for the development of novel anthelmintic compounds, but their limited amount remains a significant hurdle for further study.

## Materials and Methods

### Ethics statement

Animal experiment was approved by the Centre Val de Loire Ethics committee (Licence number APAFIS#2224-2015100917282331 v2) and carried out according to EU regulation on animal experimentation (2010/63/UE). Due to importance of dairy goat production, the *in vivo* trial was implemented on females. For sheep, we chose to focus also on ewes to achieve sufficient statistical power while limiting animal use. Although the effect on growing male lambs was not tested, our results would still be relevant to the field whereby flocks are structured around lambing ewes.

By the end of the trial, every enrolled ewe and goat were drenched with ivermectin for ewes, Oramec^®^, 2.5 mL/10 kg bodyweight, Merial, France) and fenbendazole for goats (Panacur 2.5% NDV, 10 mg/kg, MSD, France) and reintroduced back into the experimental flock for production.

### Lupin species and varieties

An initial screening for anthelmintic activity was performed on 11 varieties of lupin seeds belonging to four different species, *i*.*e*. *L*. *albus*, *L*. *angustifolius*, *L*. *luteus* and *L*. *mutabilis* that exhibited either high or low alkaloid content (Supplementary Table 1). Alkaloid-rich seeds are not commercially available and were provided by the national biobank for proteaginous plants (CRB Protéagineux, INRA, Dijon, France) as well as the LANG172 and LL049 varieties. Other commercial varieties with low alkaloid content, namely CLOVIS, ENERGY, and ORUS were provided by Jouffray-Drillaud (Lusignan, France).

### Lupin seed extract preparation

To prepare lupin seed aqueous extracts used in the initial screening, seeds were crushed into powder and incubated within water (1/5 w/w) over 48 h at 20 ± 2 °C. The aqueous extract was then filtered on cotton mesh before being frozen and lyophilised. For parasitological tests, lyophilised extracts were diluted in water at 5 mg/mL and passed through a 0.2 µm mesh filter to increase solution transparency and prevent the obstruction of the migration mesh, thereby preventing any interaction with the larval migration assay.

### Alkaloid extraction, fractionation and identification

Lupin alkaloids extraction was adapted from previous work^46^. Lupin seeds were crushed and extracted five times with HCl 0.5 M (1/6, w/w), before adjusting the pH to 12 with 40% NaOH and three subsequent extraction with CH_2_Cl_2_ (6:1, v/v). The resulting organic phases were pooled, dried with MgSO_4_ and concentrated under vacuum before three additional extractions with HCl 0.05 M (3:1, v/v). Residual traces of CH_2_Cl_2_ were eliminated by evaporation under vacuum, and the resulting aqueous alkaloid extract was freeze dried.

Compositional insights on the alkaloid fractions was subsequently inferred by ultra-high-performance thin layer chromatography (UHPLC). For UHPLC, mobile phases were solvent A 0.1% TFA in water and solvent B acetonitrile. Gradient was set by raising the acetonitrile content from 0% to 100% in 15 min and maintained for 2 min, with a flow rate of 0.8 mL.min^−1^, and oven temperature set at 40 °C. The chromatogram was monitored at 205, 220 and 305 nm and the calibration curve was derived using 5 µL of 200, 400, 600, 800 and 1,000 µg/mL lupanine perchlorate (Innosil, Poznán, Poland) solutions. Lupin aqueous maceration or alkaloid extract were solubilized in water to obtain a final concentration between 1 and 20 mg/mL as required.

To identify the alkaloidic compounds harboured by ENERGY seeds, crude alkaloids extract (1.9 g) obtained from 3.2 kg of seeds were fractionated using Centrifugal Partition Chromatography (CPC) in pHzone refining mode with a biphasic solvent system in descending mode (Methyl terbutyl ether/n-Butanol/water, 23:32:45, v/v/v). Triethylamine (29.4 mM) was used as a retainer in organic stationary phase, and HCl (3.3 mM) was used as a displacer in aqueous mobile phase. Chromatographic parameters were set as follows: flow rate 6 mL.min^−1^; rotation speed 1400 rpm; back pressure 40 bars; stationary phase retention 70%. Resulting fractions were purified using semi-preparative HPLC with the same UHPLC chain (See Supplementary Material). Isolated alkaloids were then analysed using mass spectrometry (LC-ESI-MS) as described elsewhere^47^. Compounds structure were proposed according to retention time combined with m/z and fragmentation profile by comparison with literature^48^.

### Larval migration inhibition assay test (LMIA)

Parasite material was maintained by the INRA animal parasite nematode collection^49^. Two *H*. *contortus* isolates that were. susceptible or resistant to every licensed anthelmintic compound in France (benzimidazoles, levamisole, macrocyclic lactones) were used. The fully susceptible isolate was primarily isolated in the United-Kingdom and displayed an *in vitro* levamisole EC_50_ of 1.1 µM (Automated Larval Migration inhibition Assay) and 100% Faecal Egg Count reduction *in vivo*. The multidrug resistant isolate was first isolated in South-Africa^50^ and we determined a levamisole EC_50_ of 15.58 µM and an *in vivo* Faecal Egg Reduction Test value of 31.6% (11400 eggs/g before treatment, 7800 eggs/g 14 days after levamisole treatment). This isolate is also resistant to triclabendazole (EC_50_ = 0.587 µg/ml relative to 0.022 µg/ml for the susceptible control) and ivermectin (EC_50_ = 0.055 µM; Faecal Egg Reduction Test value of 67.8%, 1450 eggs/g before treatment, 450 eggs/g 14 days after ivermectin treatment). To establish whether lupin effect was conserved across GIN species, a fully susceptible French *T*. *circumcincta* isolate^51^ was used. Its susceptibility to levamisole was confirmed *in vitro* with ALMA (IC_50_ = 1.79 µM). For every test, infective larvae were exsheathed with 0.5% hypochlorite solution and rinsed thrice with water before subsequent incubation in 5 mg/mL solutions of either crude maceration, alkaloidic fraction or non-alkaloidic fraction, levamisole (10 µM) or water, for 18 h at 20 °C. At the end of the incubation period, 100 L3 were deposited on migration plates with 20 µm mesh at 38 °C for 2 h as described elsewhere^52^. Typically, for each variety of the initial screening on 11 varieties, three replicates were made and normalized by six control observations. Dose-response assays were run with six control replicates and four replicates by concentration tested (0, 5, 7.5 and 10 mg/mL for E063 alkaloids and 0, 2, 4, 6 and 8 mg/mL for ENERGY alkaloids).

### Automated Larval Migration Assay (ALMA) for the isolated alkaloid from energy seeds

Because of the scarcity of fractionated alkaloidic compounds (2.7 to 12.8 mg), their anthelmintic effect on infective *H*. *contortus* larvae was measured using ALMA. This technique relies on larval auto-fluorescence generated through ultraviolet excitation^42,43^. Every alkaloidic compound was incubated for 4 h at 20 °C with 7,500 *H*. *contortus* infective larvae each. Larvae were then passed through a 20 μm sieve and left for a 60 s stabilization time before migration into the spectrofluorimeter cuvette. Cumulative fluorescence resulting from larval migration into the cuvette and correlating the total number of larvae^43^ was recorded during 5 min. Each set of experiment was performed in triplicate at a concentration of 250 µg/mL but for compound **2** (150 µg/mL). Levamisole 10 µM was used as a positive control.

### *Xenopus laevis* oocyte electrophysiology

The lupanine and alkaloid extracts from lupin seeds were perfused on defolliculated *X*. *laevis* oocytes (Ecocyte Bioscience, Dortmund, Germany) expressing either the *C*. *elegans* nicotine-sensitive nAChR subtype (Cel-N-nAChR, encoded by *acr-16*^53^), or its levamisole-sensitive counterpart (Cel-L-nAChR, encoded by *unc-38*, *unc-63*, *unc-29*, *lev-1* and *lev-8*^53^). In the lack of *H*. *contortus* nicotine-sensitive AChR described, the sole levamisole-sensitive nAChR subtype 1 (Hco-L-nAChR1, encoded by *unc-29*.*1*, *unc-38*, *unc-63* and *acr-8*^29^) from *H*. *contortus* was also expressed. Expression of recombinant nAChRs was achieved by microinjection of cRNAs into the oocyte cytoplasm and two-electrode voltage-clamp recordings were carried out as previously described^29^.

Acetylcholine was applied first to check for the presence of functional nAChRs and for current normalization in presence of lupanine or alkaloid extracts. Antagonist modulation of acetylcholine-elicited current was determined by pre-application of lupanine or alkaloid extracts for 10 seconds, followed by their co-application in the presence of acetylcholine.

Data were collected and analysed using pCLAMP 10.4 package (Molecular Devices, UK).

### *In vivo* testing

To establish the palatability and the *in vivo* anthelmintic effect of lupin seeds, 48 female Romane ewe and 48 Alpine dairy goats were allocated to four experimental groups each corresponding to possible meal composition and infection status combinations (**Lup-Inf**: lupin-fed and infected; **Lup-Ninf**: lupin-fed and not infected; **Conc-Inf**: concentrate-fed and infected; **Conc-Ninf**: concentrate-fed and not infected). Dietary supplementation with protein can increase host resilience to *H*. *contortus* infection^54^. Therefore, lupin- and concentrate-diets were designed to be balanced in their total energy and protein content (Supplementary Table 10) and to fulfil animal nutritional needs (a growth rate of 150 g/day for ewes and a 3.1 kg/day milk production for goats)^55^. Lupin- and concentrate-diets were similar in energy (around 1,580 and 3,600 kcal NEL, for ewes and goats respectively), proteins (around 13% and 14% CP, for ewes and goats respectively) and fibres (around 20% and 22% CF, for ewes and goats respectively).

Animals were challenged with the same susceptible *H*. *contortus* isolate as the one used for *in vitro* testing. The experimental infection was run indoor and was set up to mimic a field chronic infection. Therefore, animals were challenged (with 5,000 drug susceptible isolate *H*. *contortus* larvae), drenched 30 days after infection and remained uninfected for a 15-day washout period. Ewes were treated with ivermectin (Oramec^®^, 2.5 mL/10 kg bodyweight, Merial, France) whereas goats were treated with fenbendazole (Panacur 2.5% NDV, 10 mg/kg, MSD, France) that is licensed for dairy production. Following this first infection, animals were assumed to have developed an immune response. To mimic chronic exposure happening in the field, animals were re-infected with 5,000 *H*. *contortus* and parasitological and production traits were measured to identify any beneficial effect of lupin seeds. Blood and faecal samples were taken just before infection, at 18-, 21-, 24- and 30-days post-infection (dpi) onwards to measure haematocrit and Faecal Egg Count (FEC). Ewes were weighted at every time point and goat milk production was recorded (morning milking) at 0, 21 and 30 dpi.

To evaluate any putative effect of lupin on larval development, faecal samples (10 g) were collected at 30 dpi from the two most infected individuals, mixed together and allocated to six culture batches incubated for 12 days at 25 °C and 60% humidity). Infective third stage larvae were then counted and the ratio between observed and expected (derived from the total number of eggs used for incubation) number of larvae (larval development rate) was derived.

### Statistical tests

The inhibitory potential of each variety was computed as:

$$p=1-\frac{No.\,treated\,migrated\,larvae}{Average\,No.\,control\,migrated\,larvae}$$

This proportion was subsequently regressed upon variety and parasitic isolate, simultaneously accounting for the migration plate effect fitted as a random effect with the lme function of the R software v3.5^56^. Model diagnostics was run to check for residual normality and detect putative outliers that were discarded accordingly. Post-hoc comparisons accounting for multiple comparisons between tested effect levels were performed using a Tukey’s test implemented with the *multcomp* package v1.4-8^57^.

Dose-response data were modelled as a log-logistic regression with two parameters, *i*.*e*. the curve slope and the associated median excitatory (EC50) or inhibitory (IC50) concentration using the *drc* package v3.0-1^58^. To account for differences in maximal response values, a third parameter was added for electrophysiology data modelling.

Linear regression of FEC and haematocrit upon fixed effects (experimental group, day post infection and their interaction) and individual fit as a random effect, was implemented with the *lme* function of the R v3.5 software^56^. To account for group heterogeneity in haematocrit at the beginning of the 2^nd^ infection (FEC were 0 for every individual at 0 dpi), basal haematocrit level measured at 0 dpi was used as covariates. Average daily gain was computed for every ewe as the difference between weights at 30 and 0 dpi divided by the number of days, and recorded milk production volumes were summed for each goat as a proxy for their total volume. Both traits were regressed upon experimental group accounting for inter-individual variation. Every measured trait but FEC was normally distributed (assessed by Shapiro-Wilk’s test values of 0.85 and 0.81 for average daily gain and transformed FEC, and above 0.96 for other traits). FEC were thus applied a fourth-root transformation to correct for normality departure.

Average prepatent period length and larval development rate were compared between diets within species using a Wilcoxon’s test.

Unless stated otherwise, average estimates are reported with their standard error (s.e.m.).

## Supplementary information

Supplementary information

## Data Availability

Data have been provided as supplementary information. Code for data analysis and figure reproduction is available under a github repository (https://github.com/guiSalle/LupinAnthelmintic). Electrophysiology data are available upon requests to Dr Claude Charvet.
