# Supplementary material for: Lupin (Lupinus spp.) seeds exert anthelmintic activity associated with their alkaloid content
Source: Sci Rep. 2019 Jun 21;9:9070. doi: 10.1038/s41598-019-45654-6 (PMC6588613; doi:10.1038/s41598-019-45654-6)
Supplement: Supplementary file 1 — Supplementary information [file 41598_2019_45654_MOESM1_ESM.pdf]

Lupin (*Lupinus spp.*) seeds exert anthelmintic activity associated with their alkaloid content

Dubois O.[1,2,‡], Allanic C.[1,‡], Charvet C. L.[1], Guégnard F.[1], Février H.[1], Théry-Koné I.[2], Cortet J.[1], Koch C.[1], Bouvier F.[3], Fassier T.[3], Marcon D.[3], Magnin-Robert J.B.[4], Peineau N.[5], Courtot E.[1], Huau C.[6], Meynadier A.[6], Enguehard-Gueiffier C.[2], Neveu C.[1], Boudesocque-Delaye L.[2,†], Sallé G.[1, †,*]

[1], UMR1282 Infectiologie et Santé Publique, INRA, Université de Tours, F-37380 Nouzilly, France

[2], EA 7502 SIMBA, Université de Tours, Faculté de Pharmacie, F-37000 Tours, France

[3], UE332 La Sapinière, INRA, F-18174 Osmoy, France

[4], Agroécologie, AgroSup Dijon, INRA, Univ. Bourgogne, Univ. Bourgogne Franche-Comté, F-21000 Dijon, France

[5], Département Physiologie Animale, Université de Tours, Faculté des Sciences et Techniques, F-37000 Tours, France

[6], GenPhySE, INRA, Université de Toulouse, INPT, ENVT, F-31326, Castanet-Tolosan, France

[†], both authors contributed equally to this work

[‡], both authors contributed equally to this work

[*] corresponding author

Guillaume Sallé

Guillaume.Salle@inra.fr

Supplementary information

Table of Contents

Supplementary Table 1. Description of the 11 lupin varieties considered in the initial screening for nematicide effect 3

Supplementary Table 2. Measured inhibitory effect of 11 lupin variety extracts on Haemonchus contortus infective larvae 4

Supplementary Table 3. Measured inhibitory effect of the alkaloidic fractions recovered from alkaloidic-rich varieties on Haemonchus contortus infective larvae 5

Supplementary Table 4. Compared inhibitory potentials of alkaloidic and non-alkaloidic fractions extracted from the ENERGY and E063 varieties against drug susceptible and -resistant H.contortus and drug-susceptible T. circumcincta 6

Supplementary table 5. Lupanine base content of ENERGY and E063 alkaloid fractions quantified by UHPLC 6

Supplementary Table 6. Median inhibitory concentrations (IC_{50}) estimated for ENERGY and E063 alkaloid fractions on H.contortus infective larvae 7

Supplementary Table 7. Average migration of H. contortus larvae after exposure to ENERGY CPC-fractionated alkaloidic extracts 7

Supplementary Table 8. Estimated median excitatory concentrations (EC_{50}) for acetylcholine in absence or presence of lupanine and inhibitory concentrations (IC_{50}) of lupanine on nematode cholinergic receptors 8

Supplementary Table 9. Average production traits and dispersion for considered experimental groups at the start of the experiment 8

Supplementary Table 10. Diet compositions for considered experimental groups 9

Supplementary Table 11. Prepatent period length and larval development rate measured in infected sheep and goats 9

Figure S1. Concentration-response curves for lupanine and levamisole obtained from larval migration inhibition assay on susceptible H.contortus infective larvae 10

Figure S2: Alkaloid extraction and identification scheme for the energy lupin variety 11

Figure S3. HPLC Chromatogram of active alkaloids fractions obtained after CPC fractionation .. 12

Figure S4. Inhibitory effect of lupanine on recombinant acetylcholine receptors 13

Supplementary Table 1. Description of the 11 lupin varieties considered in the initial screening for nematicide effect

Species	Variety	Origin	Alkaloid content
L. albus	E063	Spain	High
L. albus	EGY014	Egypt	High
L. albus	EGY100	Egypt	High
L. angustifolius	LANG061	France	High
L. luteus	LL151	Portugal	High
L. mutabilis	LM261	Peru	High
L. albus	CLOVIS*	France	Low
L. albus	ENERGY*	France	Low
L. albus	ORUS*	France	Low
L. angustifolius	LANG172	Australia	Low
L. luteus	LL049	Germany	Low

*, commercially available varieties in Europe

Supplementary Table 2. Measured inhibitory effect of 11 lupin variety extracts on *Haemonchus contortus* infective larvae

Lupin variety	Alkaloid content	*H. contortus* resistance status	Average inhibitory effect	Standard deviation
CLOVIS	Low	Multidrug resistant	61.97%	5.59%
Control	Low	Multidrug resistant	5.28%	6.14%
E063	High	Multidrug resistant	78.10%	10.03%
EGY014	High	Multidrug resistant	84.67%	3.79%
EGY100	High	Multidrug resistant	81.75%	3.34%
ENERGY	Low	Multidrug resistant	75.35%	4.88%
LANG061	High	Multidrug resistant	70.80%	12.83%
LANG172	Low	Multidrug resistant	59.86%	11.76%
LL049	Low	Multidrug resistant	73.24%	5.32%
LL151	High	Multidrug resistant	63.50%	22.37%
LM261	High	Multidrug resistant	83.94%	9.12%
ORUS	Low	Multidrug resistant	58.45%	5.32%
CLOVIS	Low	Fully susceptible	33.94%	12.00%
Control	Low	Fully susceptible	2.52%	4.18%
E063	High	Fully susceptible	53.15%	9.96%
EGY014	High	Fully susceptible	95.95%	2.70%
EGY100	High	Fully susceptible	66.22%	4.68%
ENERGY	Low	Fully susceptible	40.37%	5.73%
LANG061	High	Fully susceptible	69.37%	6.80%
LANG172	Low	Fully susceptible	8.41%	9.56%
LL049	Low	Fully susceptible	45.41%	3.18%
LL151	High	Fully susceptible	50.90%	12.26%
LM261	High	Fully susceptible	92.79%	0.78%
ORUS	Low	Fully susceptible	27.98%	7.83%

Supplementary Table 3. Measured inhibitory effect of the alkaloidic fractions recovered from alkaloidic-rich varieties on *Haemonchus contortus* infective larvae

Lupin variety	*H. contortus* resistance status	Average inhibitory effect	Standard deviation
Water control	Multidrug resistant	0.00%	12.90%
E063	Multidrug resistant	81.99%	3.58%
EGY014	Multidrug resistant	72.99%	8.65%
EGY100	Multidrug resistant	73.46%	8.33%
LANG061	Multidrug resistant	69.67%	5.75%
LL151	Multidrug resistant	59.24%	10.67%
LM261	Multidrug resistant	78.20%	6.41%
Water control	Fully susceptible	0.00%	10.77%
E063	Fully susceptible	77.59%	3.95%
EGY014	Fully susceptible	61.49%	7.53%
EGY100	Fully susceptible	73.28%	3.95%
LANG061	Fully susceptible	54.31%	9.00%
LL151	Fully susceptible	52.01%	2.77%
LM261	Fully susceptible	56.03%	2.28%

Supplementary Table 4. Compared inhibitory potentials of alkaloidic and non-alkaloidic fractions extracted from the ENERGY and E063 varieties against drug susceptible and -resistant *H.contortus* and drug-susceptible *T. circumcincta*

	***H. contortus* - Drug susceptible**		***H. contortus* - Multidrug resistant**		***T. teladorsagia* - Susceptible**	
	Average	Std.	Average	Std.	mean	Std.
Water	0.17%	5.42%	-0.17%	8.42%	0.00%	13.49%
Levamisole	99.50%	1.22%	30.50%	6.38%	73.70%	6.68%
E063-Total Extract	83.00%	4.90%	28.33%	7.28%	98.00%	2.68%
E063.Alkaloids	41.17%	13.53%	40.17%	9.06%	51.00%	9.14%
E063.Non-alkaloids	37.67%	7.79%	12.50%	6.41%	21.70%	8.19%
ENERGY-Total Extract	42.67%	8.24%	48.67%	15.55%	90.20%	8.95%
ENERGY.Alkaloids	41.50%	16.56%	54.83%	9.06%	72.30%	9.79%
ENERGY.Non-alkaloids	-12.33%	15.63%	-10.00%	20.34%	6.00%	18.81%

Average inhibitory effect and corresponding standard deviations are presented for each every tested condition across *H.contortus* isolates and a susceptible *T. circumcincta* isolate. Each condition was run in triplicate. Levamisole was used at 10 µM and lyophilized extracts were used at a concentration of 5mg/mL.

Supplementary table 5. Lupanine base content of ENERGY and E063 alkaloid fractions quantified by UHPLC

**Lupin variety**	**Lupanine base (µg)**	**Extract injected (µg)**	**Percentage of Lupanine base (%)**
ENERGY	2.15 ± 0.02	10	21.5 ± 0.2
E063	2.97 ± 0.01	6	49.5 ± 0.2

Supplementary Table 6. Median inhibitory concentrations (IC_{50}) estimated for ENERGY and E063 alkaloid fractions on *H.contortus* infective larvae

		IC_{50}	Std.	Lower bound	Upper bound
Multidrug resistant *H. contortus*	E063	8.38	1.15	6.05	10.72
	ENERGY	5.59	0.47	4.64	6.55
Fully susceptible *H. contortus*	E063	9.30	0.73	7.82	10.78
	ENERGY	4.54	0.21	4.11	4.96

IC_{50} was estimated from a log-logistic regression. *Std* indicates estimated standard deviation, and lower and upper bound of the 95% confidence interval are provided.

Supplementary Table 7. Average migration of *H. contortus* larvae after exposure to ENERGY CPC-fractionated alkaloidic extracts

Isolate	Fraction	Percentage of migrating larvae	Standard deviation
	Crude alkaloid extract	43.8	11.7
	F1	91.7	17.4
	F2	0.8	1.4
	F3	2.5	2.5
	F4	120.7	24.5
	F5	58.7	5.2
Multidrug-resistant	F6	58.7	20.2
	F7	38.8	5.2
	F8	14.9	5.0
	F9	22.3	5.0
	F10	6.6	3.8
	Levamisole (10 µM)	39.7	8.9
	Negative control	100.0	19.9
	Crude alkaloid extract	49.3	2.1
	F1	91.4	24.7
	F2	15.0	2.1
	F3	5.7	4.5
	F4	90.0	9.3
	F5	80.0	24.4
Fully susceptible	F6	74.3	5.0
	F7	33.6	6.2
	F8	17.9	2.5
	F9	15.7	1.2
	F10	6.4	3.7
	Levamisole (10 µM)	12.1	5.4
	Negative control	100.0	12.6

Results of a Larval Migration Inhibition Assay are provided for ten fractions obtained after CPC-fractionation of ENERGY alkaloidic extracts and corresponding crude extract. Percentages and standard deviations were estimated from three replicates. Water was used a negative control. Fractions were tested at a concentration of 2.5 mg/mL, but fractions 1 and 8 (0.625 mg/mL).

Supplementary Table 8. Estimated median excitatory concentrations (EC_{50}) for acetylcholine in absence or presence of lupanine and inhibitory concentrations (IC_{50}) of lupanine on nematode cholinergic receptors

	Cel-N-AChR	Cel-L-AChR	Hco-L-AChR-1
Acetylcholine alone			
EC_{50} (µM)	21.7 ±0.9	19.6 ±1.7	4.8 ±0.5
n	12	11	10
Acetylcholine with Lupanine (300 µM)			
EC_{50} (µM)	80.6 ±27.5	99.1 ±47.8	28.6 ±8.4
n	5	6	8
Lupanine			
IC_{50} (µM)	116.5 ±9.7	548.8 ±64.1	539.9 ±90.2
n	5	5	5

EC_{50} of acetylcholine and IC_{50} of lupanine and standard deviation are reported for *Caenorhabditis elegans* nicotine- (Cel-N-AChR) and levamisole-sensitive (Cel-L-AChR) receptors, and *H. contortus* levamisole-sensitive AChRs (Hco-L-AChR-1). *n* refers to the number of *X. laevis* oocytes measured in each case.

Supplementary Table 9. Average production traits and dispersion for considered experimental groups at the start of the experiment

Species	Trait	Lup-Inf	Lup-Ninf	Conc-Inf	Conc-Ninf
Goat	Milking rank	1.9 ± 1.1 [1;4]	2.5 ± 1.3 [1;5]	2.1 ± 1.0 [1;4]	2.3 ± 1.4 [1;6]
	Milking stage (days)	103 ± 18 [71;129]	116 ± 16 [84;131]	102 ± 20 [68;122]	107 ± 20 [77;128]
	Milking volume (L)	1.8 ± 0.3 [1.4;2.2]	1.6 ± 0.5 [0.8;2.4]	2.1 ± 0.4 [1.5;2.6]	1.9 ± 0.4 [1.4;2.5]
Sheep	Age (days)	124 ± 2.1 [121;129]	125 ± 2.0 [122;129]	124 ± 2.3 [121;128]	123 ± 2.5 [120;129]
	Weight (Kg)	36.1 ± 4.2 [26.8;41.5]	37.3 ± 3.1 [31.1;42.7]	36.1 ± 3.3 [32.7;43.6]	36.4 ± 3.3 [30.6;42.5]

Summary statistics of ewes and goats production traits are listed for the four considered experimental groups (Lup-Inf: lupin-fed and infected; Lup-Ninf: lupin-fed and not infected; Conc-Inf: concentrate-fed and infected; Conc-Ninf: concentrate-fed and not infected). Statistics are given as "mean ± standard deviation [minimum ; maximum]" and computed from 12 individuals in each case.

Supplementary Table 10. Diet compositions for considered experimental groups

Species	Group	Ingredient	Quantity
Sheep	Conc-Inf/Conc-Ninf	Commercial concentrate	0.51
		Hay	0.66
	Lup-Inf/Lup-Ninf	Lupin	0.25
		Barley	0.17
		Hay	0.66
Goat	Conc-Inf/Conc-Ninf	Rapeseed meal	0.63
		Commercial concentrate	0.22
		Hay	2
	Lup-Inf/Lup-Ninf	Lupin	0.45
		Commercial concentrate	0.22
		Hay	2

Ingredients quantity are given in kg of organic matter/individual/day for each dietary type in both species. Lup-Inf: lupin-fed and infected; Lup-Ninf: lupin-fed and not infected; Conc-Inf: concentrate-fed and infected; Conc-Ninf: concentrate-fed and not infected.

Supplementary Table 11. Prepatent period length and larval development rate measured in infected sheep and goats

Species	Infected group	Prepatent period (days)	Larval development rate (%)
Ewe	Lup-Inf	23.3 ± 2.9	53.3 ± 25.5
	Conc-Inf	24.8 ± 4.3	55.2 ± 22.3
Goat	Lup-Inf	24.2 ± 3.5	32.0 ± 15.7
	Conc-Inf	25.3 ± 4.6	34.3 ± 21.9

Prepatent period was computed as the average number of days before the onset of egg excretion within group (n = 12). Larval development rate was estimated from 6 replicates. Results are reported as “mean ± standard deviation”. Lup-Inf: lupin-fed and infected; Conc-Inf: concentrate-fed and infected.

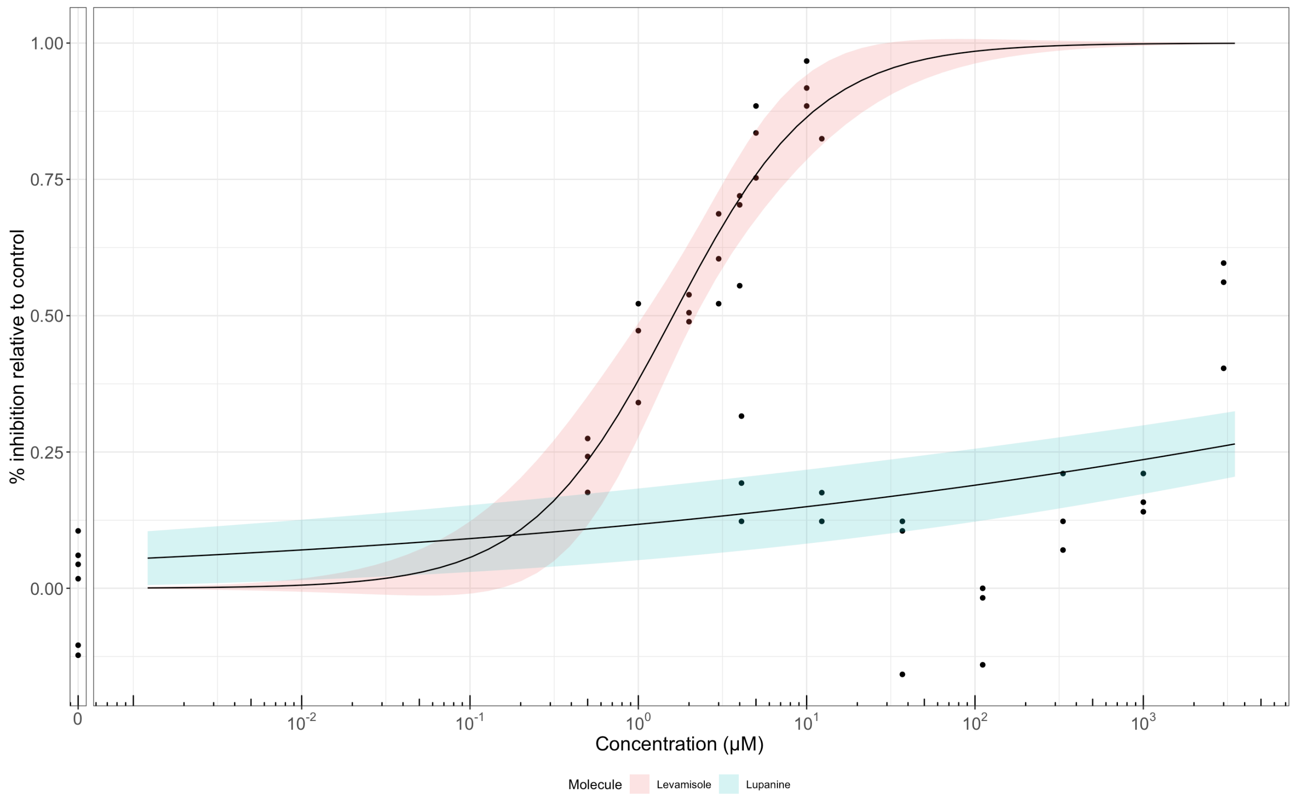

Figure S1. Concentration-response curves for lupanine and levamisole obtained from larval migration inhibition assay on susceptible *H.contortus* infective larvae

Plot shows the inhibited fraction of larvae relative to control for Lupanine and levamisole concentration ranging between 0 and 3 mM. Solid line stands for the fitted log-logistic regression curve and shaded area indicates 95% confidence interval.

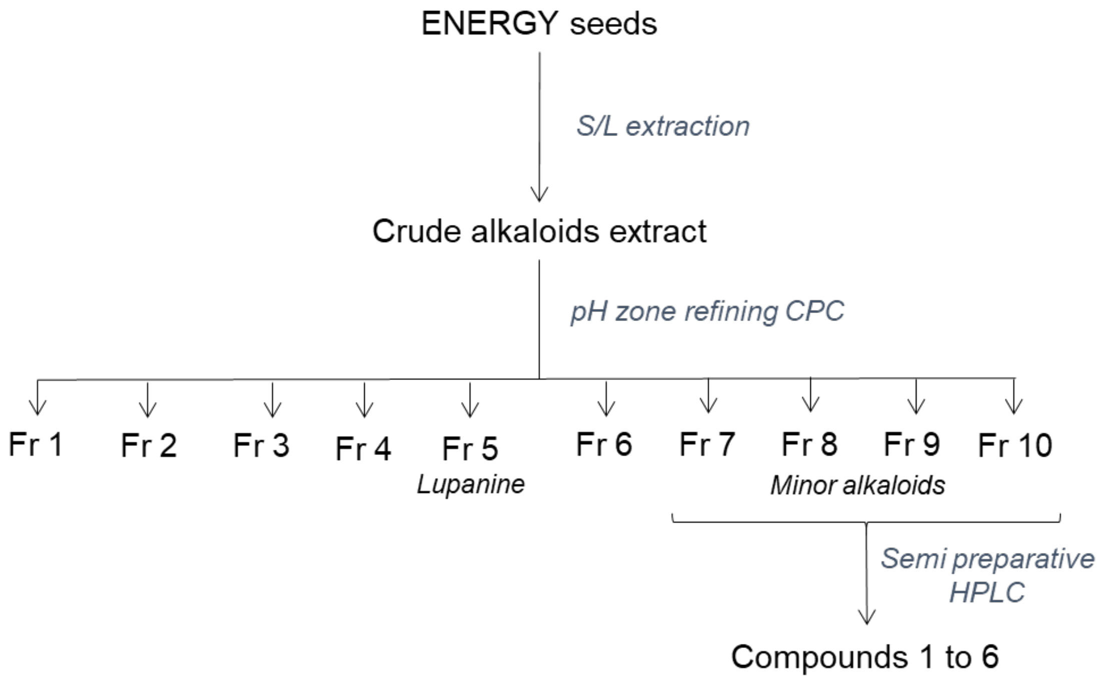

Figure S2: Alkaloid extraction and identification scheme for the energy lupin variety

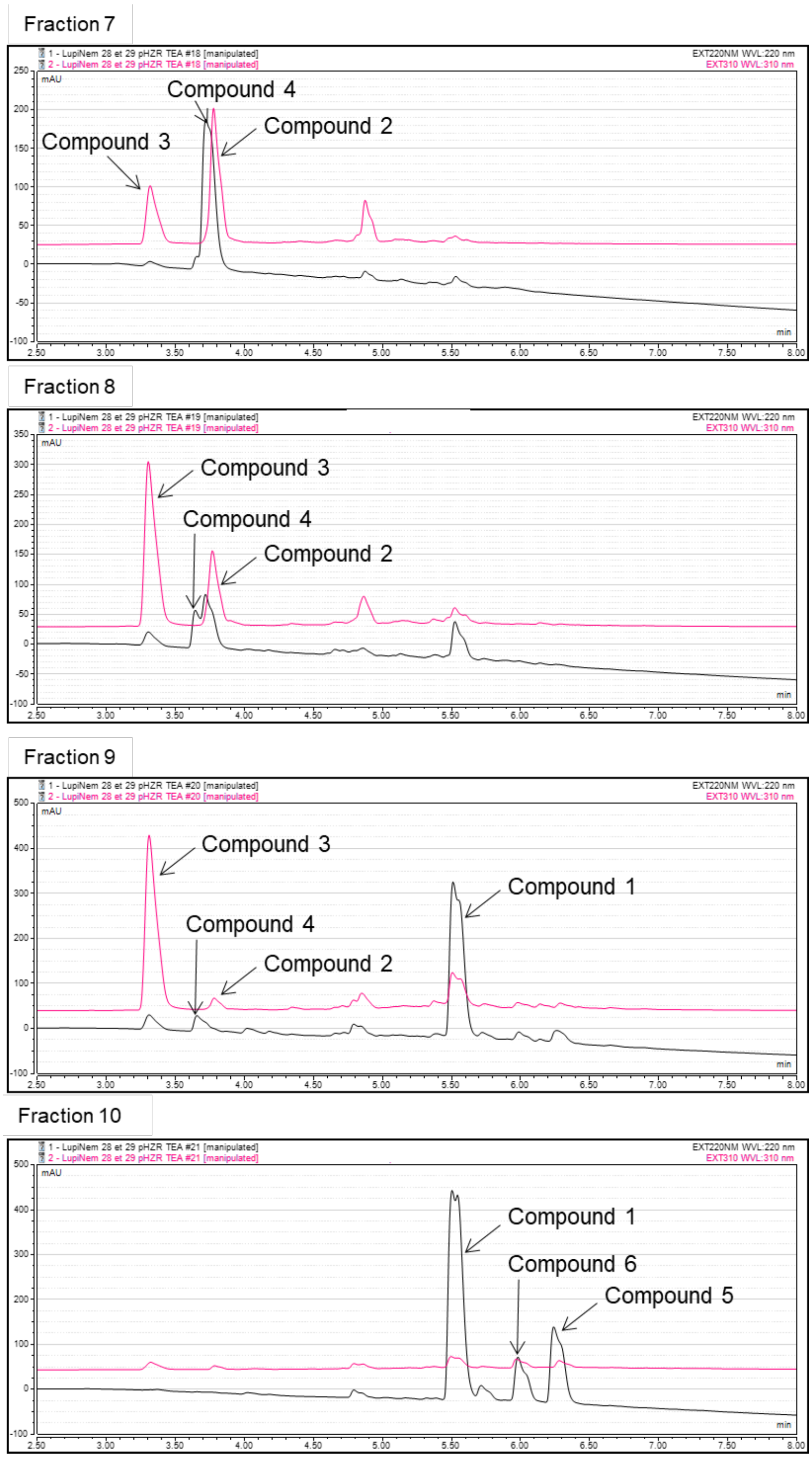

Figure S3. HPLC Chromatogram of active alkaloids fractions obtained after CPC fractionation

HPLC profile at 220 nm (black) and 310 nm (pink) of fractions 7 to 10 were represented here.

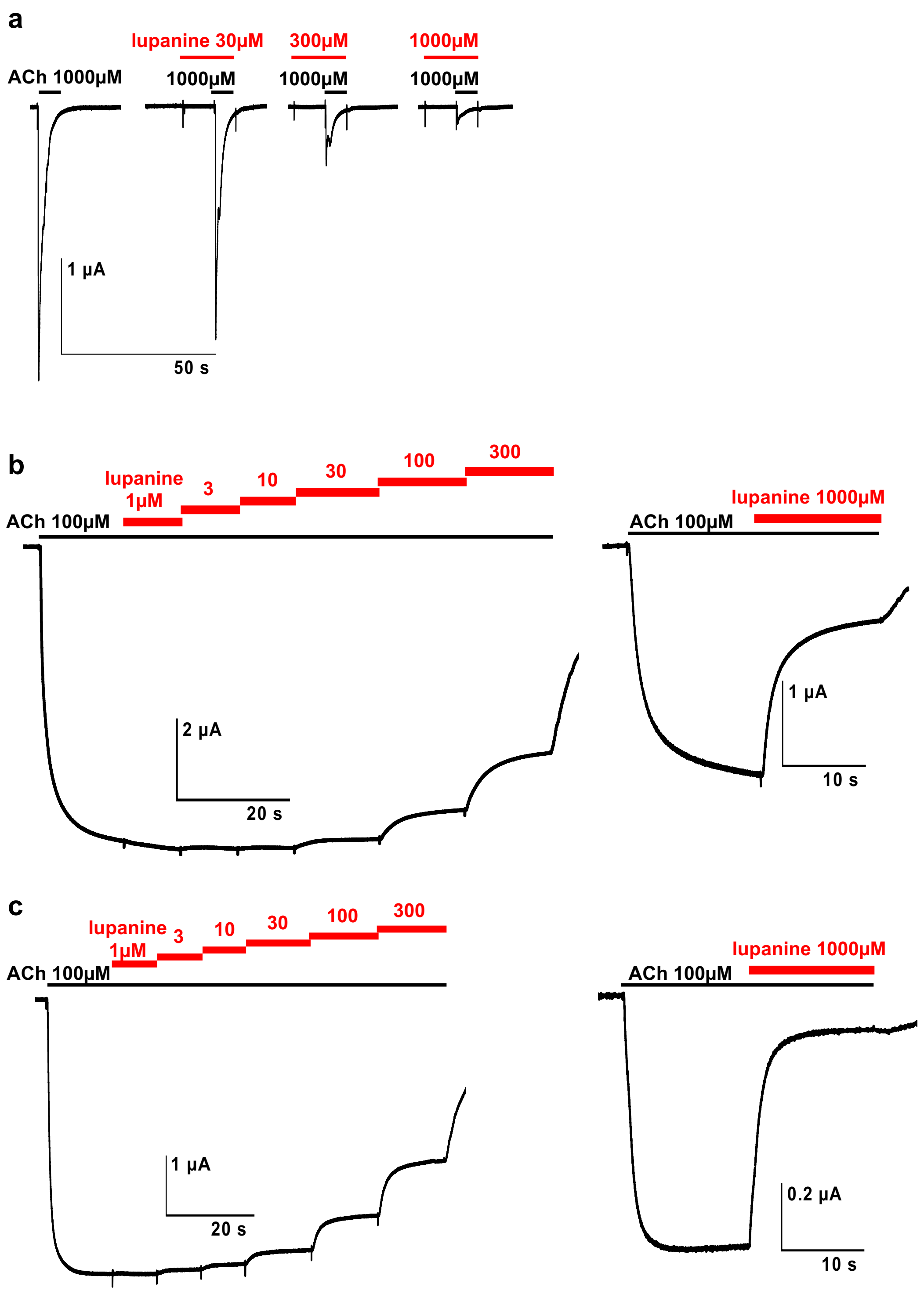

Figure S4. Inhibitory effect of lupanine on recombinant acetylcholine receptors

Representative recording traces of ACh response (black bar) in the presence of increasing concentrations of lupanine (red bars) on the *C. elegans* N-AChR (a) and L-AChR (b) and the *H. contortus* Hco-L-AChR-1 (c) are plotted.
